# Natural Histogel-Based Bio-Scaffolds for Sustaining Angiogenesis in Beige Adipose Tissue

**DOI:** 10.3390/cells8111457

**Published:** 2019-11-18

**Authors:** Margherita Di Somma, Wandert Schaafsma, Elisabetta Grillo, Maria Vliora, Eleni Dakou, Michela Corsini, Cosetta Ravelli, Roberto Ronca, Paraskevi Sakellariou, Jef Vanparijs, Begona Castro, Stefania Mitola

**Affiliations:** 1 Department of Molecular and Translational Medicine, University of Brescia, 25121 Brescia, Italy; m.disomma88@gmail.com (M.D.S.); elisabetta.grillo@unibs.it (E.G.); mvliora@gmail.com (M.V.); michela.corsini@unibs.it (M.C.); cosetta.ravelli@unibs.it (C.R.); roberto.ronca@unibs.it (R.R.); 2 Histocell, S.L.Parque Tecnológico 801A, 2o 48160 Derio—BIZKAIA, Spain; wandertschaafsma@gmail.com (W.S.); bcastro@histocell.com (B.C.); 3 FAME Laboratory, Department of Exercise Science, University of Thessaly, 38221 Trikala, Greece; sakellariou.elvina@gmail.com; 4 Laboratory of Cell Genetics, Department of Biology, Faculty of Science and Bioengineering Sciences, Vrije Universiteit Brussel, 1050 Brussels, Belgium; Eleni.Dakou@vub.be; 5 Department of Human Physiology, Faculty of Physical Education and Physical Therapy, Vrije Universiteit Brussel, 1050 Brussels, Belgium; Jef.Vanparijs@vub.be

**Keywords:** angiogenesis, adipose derived mesenchymal cells, Histogel

## Abstract

In the treatment of obesity and its related disorders, one of the measures adopted is weight reduction by controlling nutrition and increasing physical activity. A valid alternative to restore the physiological function of the human body could be the increase of energy consumption by inducing the browning of adipose tissue. To this purpose, we tested the ability of Histogel, a natural mixture of glycosaminoglycans isolated from animal Wharton jelly, to sustain the differentiation of adipose derived mesenchymal cells (ADSCs) into brown-like cells expressing UCP-1. Differentiated cells show a higher energy metabolism compared to undifferentiated mesenchymal cells. Furthermore, Histogel acts as a pro-angiogenic matrix, induces endothelial cell proliferation and sprouting in a three-dimensional gel in vitro, and stimulates neovascularization when applied in vivo on top of the chicken embryo chorioallantoic membrane or injected subcutaneously in mice. In addition to the pro-angiogenic activity of Histogel, also the ADSC derived beige cells contribute to activating endothelial cells. These data led us to propose Histogel as a promising scaffold for the modulation of the thermogenic behavior of adipose tissue. Indeed, Histogel simultaneously supports the acquisition of brown tissue markers and activates the vasculature process necessary for the correct function of the thermogenic tissue. Thus, Histogel represents a valid candidate for the development of bioscaffolds to increase the amount of brown adipose tissue in patients with metabolic disorders.

## 1. Introduction

Obesity represents a major health problem associated with increased mortality and co-morbidities, including many metabolic diseases [1]. Obesity is characterized by an increase in adipose mass due to increased energy intake, decreased energy expenditure, or both. Several elements including lifestyle, environmental, neuro-psychological, genetic, and epigenetic factors contribute to increase the energy intake (calories) and to reduce the energy expenditure (metabolic and physical activity). The treatment options for patients with severe obesity are the modification of lifestyle, limiting the intake of total fats and sugars, increasing consumption of fruits, vegetables, and whole grains and practicing regular physical activity or pharmacological treatments currently available in the market, which are commonly associated with severe side effects. Indeed, increased risk of psychiatric disorders and non-fatal myocardial infarction or stroke have been described in pharmacologically treated patients [2,3,4]. In the treatment of obesity, only 1% of patients receive bariatric surgery despite its safety and the better outcomes achieved [5]. Therefore, several groups are working on the development of different and new therapeutic approaches [3]. The increase of energy consumption through the increase of metabolism and thermogenesis of the adipose tissue may be a valid alternative [6].

Adipose tissue (AT) contains adipocytes and pre-adipocytes surrounded by stromal cells (fibroblasts, endothelial cells, macrophages), which make the adipose tissue the most plastic organ in the human body. AT is the major organ that controls the overall energy homeostasis in a living organism, storing the superabundant nutrients in the form of triglycerides, and suppling the nutrients to other tissues through lipolysis [7]. Two different types of adipose tissue have been described in mammalians. White adipose tissue (WAT) stores energy, while brown adipose tissue (BAT) is specialized for energy expenditure. The cellular structure of these tissues well reflects their biological functions: WAT contains adipocytes with a single large lipid droplet and few mitochondria, while BAT cells are characterized by the presence of several small lipid droplets and many mitochondria expressing uncoupling proteins (UCPs). UCP-1 contributes to energy loss as heat; in particular, it controls the dissipation as heat of the proton gradient produced by the mitochondria respiration chain. In humans, BAT mass declines with age, and it is less active in obese patients. The adipose tissue (AT) can be considered an endocrine organ, as both WAT and BAT secrete many cytokines, hormones, and adipokines. Recently, an additional/intermediate AT cell type termed “beige” has been described. Beige adipocytes sporadically reside with white adipocytes and emerge in response to certain environmental cues [8]. Under specific stimuli, beige adipocytes can exert BAT-like or WAT-like functions. Although beige adipocytes express a low level of UCP-1, they retain a remarkable ability to activate the expression of this gene powerfully and to turn on a robust program of respiration and energy expenditure that is equivalent to that of classical brown fat cells [8,9]. These cells represent a cellular mechanism to provide flexibility in adaptive thermogenesis and metabolism. Thus, the possibility to generate and control the amount of beige adipocytes may represent an alternative therapy for obese patients.

Blood vessels play a key role in the regulation of AT behavior. AT is the most plastic organ in the human body, subjected to continuous expansion and regression. This plasticity requires constant growth, regression, and remodeling of blood vessels, under the control of several metabolites secreted by AT itself. The adipose vasculature supports AT in multiple manners. The vascular network provides nutrients and oxygen, which are essential for tissue maintenance, and removes metabolic products of AT. Moreover, it exports the AT derived growth factors, adipokines, and cytokines from AT to body tissues, regulating physiological functions via an endocrine mechanism [10]. Furthermore, similar to mesenchymal cells [11], the adipocyte precursor cells within WAT and BAT express VEGF-A, promoting the angiogenic process and endotheliogenesis through VEGFR2 signaling [12].

Tissue engineering represents an interdisciplinary approach to regenerate damaged tissues, instead of replacing them, by developing biological substitutes that improve or restore tissue functions. Tissue engineering can help to boost human metabolism through the integration of cell biology and biomaterial sciences [13]. With particular attention to adipose tissue, tissue engineering is a promising approach to improve energy balance and metabolic homeostasis controlling the amount of beige cells [14,15]. Several biological or synthetic scaffolds have been developed to support and/or promote tissue regeneration or organ repair [16,17,18,19,20]. All the scaffolds share some characteristics including biocompatibility, neglectable immunoreactivity, and suitability for cell growth. Furthermore, scaffolds should be biodegradable, and their derivates should not be toxic to the body.

Here, we tested the ability of a novel bioscaffold able to promote both the differentiation of mesenchymal cells into beige adipose tissue and to sustain the angiogenic process. The Histogel-alginate scaffold promotes adipose tissue derived stem cell (ADSC) differentiation into adipocytes expressing PPARγ, PdK4, and UCP1 proteins supporting vessel recruitment and growth. Our results suggest that Histogel based scaffolds may represent good candidates for the development of scaffolds aimed at regulating energy expenditure in obese patients.

## 2. Materials and Methods

### 2.1. Hyaluronan Analysis

Commercial low, high molecular weight HA was electrophoretically analyzed on 1% agarose gel and stained with Stains-All solution HA (25 mg in 500 mL ethanol:water 50:50) overnight in the dark. Then, agarose gels were de-stained with water. The same protocol was used to explore the amount and the molecular weight of HA in Histogel preparations.

### 2.2. Cell Cultures and Differentiation

ADSCs were isolated from lipoaspirate (Histocell, Spain, in compliance with Certification of Laboratory 4269-E for the manufacture of research drugs (cell therapy products), Spanish Agency of Drugs and Medical Devices (Agencia Española de Medicamentos y Productos Sanitarios, AEMPS) and were cultured in DMEM, complemented with Glutamax, penicillin/streptomycin, 10% FBS, and gentamicin sulfate (identified as basal medium). ADSCs were differentiated for 15 days in basal medium in the presence of 20 nM insulin (Sigma, St. Louis, MO, USA), 5 µM dexamethasone (Sigma), 125 µM indomethacin (Sigma), 1 nM triiodothyronine (T3) and 0.5 mM 3-isobutyl-1-methylxanthine (IBMX). Lipid vesicles formed starting from Day 6 of differentiation [21]. Human umbilical vein endothelial cells (HUVECs) were isolated from umbilical cords from healthy informed volunteers and used at early passages (I–IV). Cells were grown on culture plates coated with Porcine Gelatin Type I, in M199 medium supplemented with 20% FCS, endothelial cell growth factor (10 µg/mL), and porcine heparin (100 µg/mL) [22].

### 2.3. Identification of Angiogenic Factors

Pro- and anti-angiogenic molecules released by ADSCs and ADSC derived beige cells were analyzed using the Human Angiogenesis Antibody Array (R&D System) according to the manufacturer’s instructions.

### 2.4. Quantitative RT-PCR

The expression of brown adipocyte markers was analyzed by RT-PCR. Briefly, total RNA was isolated using TRIzol reagent (Invitrogen, Carlsbad, CA, USA) according to the manufacturer’s instructions from 3 independent differentiation experiments. Two micrograms of total RNA were retro-transcribed with M-MLV reverse transcriptase (Invitrogen, Carlsbad, CA, USA) using random hexaprimers in a final 20 μL volume. Quantitative RT-PCR was performed using the iTaq^TM^ Universal SYBR^®^ Green Supermix (Bio-Rad, Hercules, CA, USA). Each PCR reaction was performed in triplicate on one plate, and fluorescence data were recorded using a Viia7 Real Time PCR System (Thermo Fisher Scientific, Waltham, MA, USA). Relative expression ratios were calculated by the Relative Expression Software Tool. The mRNA expression levels of target genes were normalized to the level of GAPDH transcript.

The following specific primers were used: Hs UCP1_Fw CGCAGGGAAAGAAACAGCAC; Hs UCP1_Rv TTCACGACCTCTGTGGGTTG; HsPdk4_FwATTTAAGAATGCAATGCGGGC; HsPdk4_RvACACCACCTCCTCTGTCTGA; Hs PPARγ_Fw CCGTGGCCGCAGAAATGA; Hs PPARγ_Rv TGATCCCAAAGTTGGTGGGC; MmCD31 FwAAGCCAAGGCCAAACAGA; Mm_CD31_Rv GGGTTTTACTGCATCATTTCC; Mm_CD45_Fw TATCGCGGTGTAAAACTCGTCAA; Mm_CD45_Rv GCTCAGGCCAAGAGACTAACGTT; Hs GAPDH_Fw GAAGGTCGGAGTCAACGGATT; Hs_ GAPDH_Rv TGACGGTGCCATGGAATTTG.

### 2.5. Mitochondrial Activity

Mitochondrial activity was monitored with Seahorse XFe24 instrument technology analysis. Oxygen consumption rate (OCR) (pmol/minute) was monitored over time before and after sequential injection of oligomycin (100 µM), carbonyl cyanide p-trifluoromethoxy-phenylhydrazone (FCCP, 100 µM), and rotenone/antimycin A (50 µM) inhibitors (which inhibit ATPase, the proton gradient, and complex I/III, respectively). Thus, ATP-linked respiration and maximal respiration were measured. Extracellular acidification rate (ECAR; mpH/minute) was also measured to observe glycolytic capability. The colorimetric 3-(4,5-dimethylthiazol-2-yl)-2,5-diphenyltetrazolium bromide (MTT) test was used to analyze the effect of isoproterenol and norepinephrine on the metabolic activity of cells, using the reducing ability of ubiquinone and CyrC and B of the mitochondrial electron transport system. For this, differentiated ADSCs were treated with 10 µM isoproterenol or 1 mM norepinephrine for 24 h. Then, cells were incubated in the presence of 0.2 mg/mL of MTT substrate for 1 h. The amount of metabolized formazan was measured by recording absorbance at 570 nm using a plate reader spectrophotometer (ELx-800 Bio-Tec Instrument).

### 2.6. EC Sprouting Assay

The collagen gel invasion assay was performed on HUVEC spheroids [23]. Briefly, spheroids were prepared in 20% methylcellulose medium, embedded in collagen gel or collagen gel:Histogel (1:5) in the absence or in the presence of ADSCs or differentiated cells. The formation of radially growing cell sprouts was observed during the next 24 h. Sprouts were counted and photographed using an Axiovert 200M microscope equipped with an LD A PLAN 20X/0,30PH1 objective (Carl Zeiss, Oberkochen, Germany).

### 2.7. In Vitro Angiogenesis Assay

Wells of µ-Slide Angiogenesis chambers (Ibidi, Martine Marne, Germany) were coated with a 0.8 mm thick layer of gel matrix by adding 10 µL of Cultrex Reduced Growth Factor Basement Membrane Matrix. After gel polymerization, 5000 HUVECs were seeded in M199 added with 5% FCS and treated with conditioned medium of undifferentiated or differentiated ADSCs. Cell viability was confirmed using calcein-AM. In live cells, the non-fluorescent calcein-AM is converted into green-fluorescent calcein. Calcein-AM is a permeant dye. After 5 h, samples were photographed using an inverted Axiovert 200 M epifluorescence microscope equipped with an LD A PLAN 20X/0,30PH1 objective (Carl Zeiss, Oberkochen, Germany). Images were analyzed using ImageJ software with the Angiogenesis plugin to detect total closed structures [24].

### 2.8. Immunofluorescence Analysis

Cells were seeded on glass coverslips and fixed in 4.0% paraformaldehyde (PFA)/2.0% sucrose in PBS, permeabilized with 0.5% Triton-X100, and saturated with goat serum in PBS. Then, cells were incubated with UCP-1 (sc-6529, SantaCruz, CA, USA), PPARγ (sc-7273, SantaCruz), and ACRP30 (sc-26497, SantaCruz) antibodies. Cells were analyzed using a Zeiss Axiovert 200M epifluorescence microscope equipped with a Plan-Apochromat 63X/1.4 NA oil objective.

### 2.9. CAM Assay

Alginate beads containing PBS, or 100 ng of recombinant human VEGF-A, or 5% of Histogel, or 30,000 cells were placed on the chicken chorioallantoic membrane (CAM) of fertilized white Leghorn chicken eggs at Day 11 of incubation [25]. After 72 h, newly formed blood vessels converging toward the implant were counted at 5× magnification using an STEMI SR stereo-microscope equipped with an objective f of 100 mm with adapter ring 475,070 (Zeiss, Oberkochen, Germany).

### 2.10. Murine Angiogenic Assay

All the procedures involving mice and their care conformed to institutional guidelines that complied with national and international laws and policies (EEC Council Directive 86/609, OJ L 358, 12 December 1987). Seven-week-old C57BL/6 mice (Charles River Laboratories International, Inc., Wilmington, MA, USA) were injected subcutaneously with 400 µL of 5% alginic acid (Sigma) containing PBS or 500 ng of VEGF-A, in the absence or in the presence of 5% Histogel solution. One week after injection, mice were sacrificed, and plugs were harvested and processed for RT-qPCR as previously described [26]. The mRNA expression levels of murine CD31 and CD45 were normalized to the levels of human GAPDH. Data are expressed as relative expression ratios (ΔΔCt—fold increase) using one PBS plug as the reference.

### 2.11. Data Representation and Statistical Analyses

Data are expressed as the mean ± SEM. Statistical analyses were performed using one-way ANOVA followed by Bonferroni’s test or Student’s *t*-test. The indicated *p*-value was set as statistically significant.

## 3. Results

### 3.1. Histogel Is a Pro-Angiogenic Bio-Scaffold

**Figure 1:**
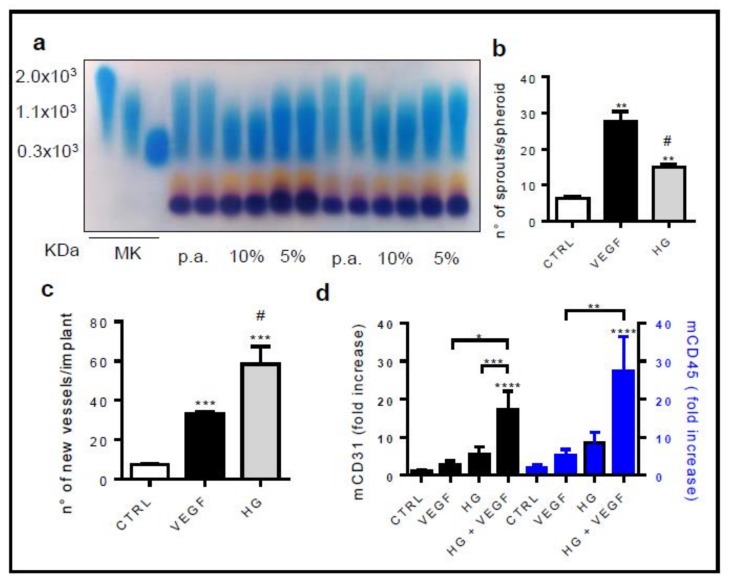
Histogel modulates the angiogenic response. (**a**) Five percent and 10% of two different Histogel preparations were electrophoretically analyzed on agarose gel and stained with Stains-All solution. p.a., pre-autoclaved. Three different standard molecules (2 × 10^3^, 1.1 × 10^3^, 300 kDa) were used as markers of molecular weight (MK). (**b**) HUVEC spheroids were embedded in collagen or in collagen:5% Histogel 1:1 (HG) gels. Fifty nanograms per milliliter of VEGFA_165_ were used as the positive control. The formation of radially growing sprouts was evaluated after 24 h of incubation. Data are the mean ± SEM of three independent experiments (** *p* < 0.001 vs. CTRL; # *p* < 0.001 vs. VEGF, one-way ANOVA followed by Bonferroni’s test versus the control). (**c**) Alginate beads containing vehicle, or 100 ng of VEGFA_165_, or 5% Histogel (*v*/*v* 1:1) were implanted on the top of chick embryo chorioallantoic membrane (CAM) at Day 11 of development. After 72 h, newly formed blood vessels converging toward the implant were counted in ovo at 5× magnification using an STEMI SR stereomicroscope equipped with an objective f equal to 100 mm with adapter ring 475,070 (Carl Zeiss). Data are the mean ± SEM (n = 6–8) (*** *p* < 0.0001 vs. control; # *p* < 0.0001 vs. VEGF, one-way ANOVA followed by Bonferroni’s test versus the control). (**d**) Five percent of liquid alginic acid was mixed with 1.0 µg/mL VEGFA_165_ in the absence or in the presence of *v*/*v* 1:1 of 5% Histogel and injected subcutaneously into the flank of C57BL/6 mice. Plugs with vehicle alone were used as negative controls (CTRL). One week after injection, plugs were harvested. CD31 and CD45 mRNA expression levels were measured by RT-qPCR. Data are the mean ± SEM (n = 10) and are expressed as relative expression ratios (ΔΔCt – fold increase) using one vehicle plug as the reference. * *p* < 0.05; ** *p* < 0.01; *** *p* < 0.005; **** *p* < 0.001, one-way ANOVA followed by Bonferroni’s test versus the control.

**Figure A1:**
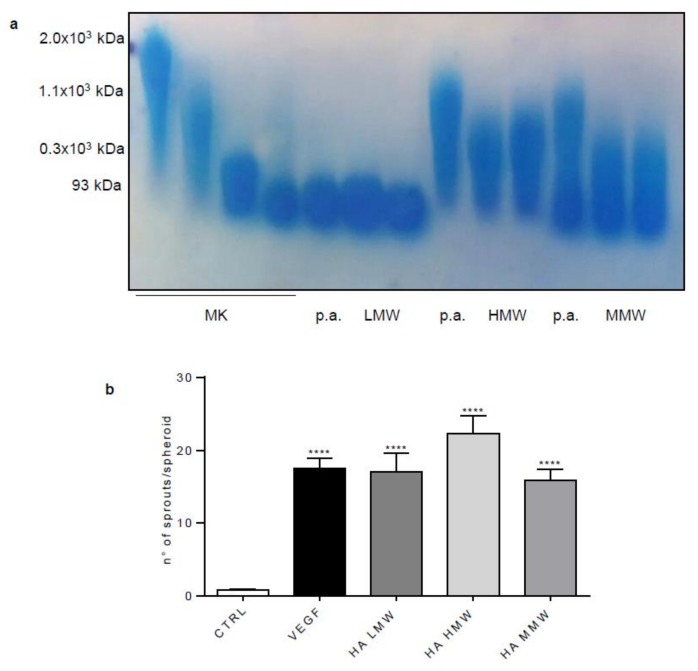
(**a**) Commercial low, high molecular weight HA was electrophoretically analyzed on agarose gel and stained with Stains-All solution. p.a. means pre-autoclaved. Three different standard molecules (2 × 10^3^, 1.1 × 10^3^, 300 kDa) were used as markers of molecular weight (MK). (**b**) HUVEC spheroids embedded in collagen gel were stimulated with commercial HA. The formation of radially growing sprouts was evaluated after 24 h of incubation. Data are the mean ± SEM of three independent experiments (** *p* < 0.001, one-way ANOVA followed by Bonferroni’s test versus the control).

Histogel is a natural mixture of glycosaminoglycans including, among others, high grade hyaluronic acid (HA) and chondroitin sulfate obtained from the Wharton jelly found in umbilical cords of animal origin [20]. Histogel modulates inflammation, induces the release of extracellular matrices (ECM), and supports cell recruitment and growth [20]. On these bases, Histogel represents a suitable scaffold to drive cell differentiation. Since hyaluronan is a mix of molecules with different masses and the molecular weight confers different biological properties to hyaluronan preparations, a first set of experiments was performed to compare the amount and the molecular weight of hyaluronan contained in different Histogel preparations. Two different Histogel preparations were analyzed, pre-autoclaved (p.a.) or not, on agarose gel, and stained with Stains-All reagent. The amount of HA was similar in both analyzed batches, and it was around 76–80%. Moreover, the ratio between high and low molecular weight molecules remained approximately constant. Of note, this ratio is not affected by the autoclave sterilization cycles used for the preparation of 5% Histogel working dilutions (Figure 1a). Next, we tested the proangiogenic activity of Histogel preparation in several in vitro angiogenesis assays. Angiogenesis is a multistep process starting with the activation of endothelial cells (ECs) and the degradation of ECM of the basal membrane. Then, ECs invade the surrounding tissue, proliferate, and reorganize themselves in capillary-like structures. Among the variety of in vitro, ex vivo, and in vivo assays that mimic the individual aspects of the angiogenic cascade, in vitro models of angiogenesis represent cost effective and rapid tools for testing angiogenic compounds. In particular, the use of 3D culture techniques able to recapitulate all steps of endothelial capillary sprout formation may serve as an effective strategy for these purposes. 3D endothelial cell spheroids were embedded in collagen or collagen:5% Histogel (1:1 ratio) gels. VEGF-A was used as the positive control and as the reference for the angiogenic activity. Figure 1b shows that, in keeping with the pro-angiogenic activity of commercial high molecular weight HA (Figure A1a,b), Histogel stimulates the formation of endothelial cell sprouts from aggregates of HUVEC cells embedded in a 3D gel. To assess the in vivo pro-angiogenic activity, Histogel loaded alginate beads were implanted onto the chicken embryo CAMs at 11 days of development. After 72 h, a robust angiogenic response was observed around the Histogel implants when compared to alginate or VEGF engrafted embryos (Figure 1c). The number of vessels converging towards the pellets was equal to 7.4 ± 0.6, 33.1 ± 1.0, and 58.3 ± 9.1 for alginate, VEGF, and Histogel implants, respectively. In keeping with these observations, Histogel modulates the recruitment of CD45^+^ cells and the consequent pro-angiogenic response when injected subcutaneously in mice (Figure 1d). All these data suggest that Histogel is endowed with stronger pro-angiogenic activity if compared to VEGF-A. The pro-angiogenic ability of Histogel makes it a suitable candidate for the development of a bioscaffold for BAT differentiation.

### 3.2. ADSCs Differentiate in Beige Adipocytes

**Figure 2:**
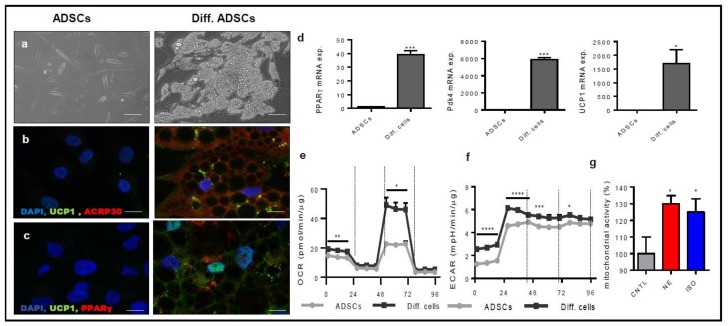
ADSCs differentiate into beige-like adipocytes. (**a**) The morphology of ADSCs and ADSC derived beige cells (Diff.) was analyzed at Day 15 of differentiation (Scale bar 100 μm). (**b**,**c**) Immunofluorescent detection of expression levels of UCP-1 (green) and ACRP30 (red) (**b**) and PPARγ (red) (**c**). Nuclei were counterstained with DAPI. (Scale bar 10 μm). (**d**) PPARγ, Pdk4, and UCP1 mRNA expression levels were measured by RT-qPCR analysis. Data are the mean ± SEM (n = 6) and are expressed as relative expression ratios (ΔΔCt – fold increase). (**e**,**f**) Mitochondrial energy metabolism was measured using the Agilent Seahorse Cell Mito Stress Test. The oxygen consumption rate (OCR) (**e**) and extracellular acidification rate (ECAR) (**f**) of ADSCs and ADSC derived beige cells was recorded before and after treatment with 10 μM oligomycin, 10 μM carbonyl cyanide p-trifluoromethoxy-phenylhydrazone (FCCP), and 5 μM rotenone/antimycin A. Data were analyzed according to the Agilent Seahorse XF Cell Mito Stress Test Report Generator. (**g**) Mitochondrial activity of ADSC derived beige cells was measured by the MTT test in the absence or in the presence of norepinephrine and isoproterenol. * *p* < 0.05; ** *p* < 0.01; *** *p* < 0.005; **** *p* < 0.001, one-way ANOVA followed by Bonferroni’s test versus the control.

**Figure A2:**
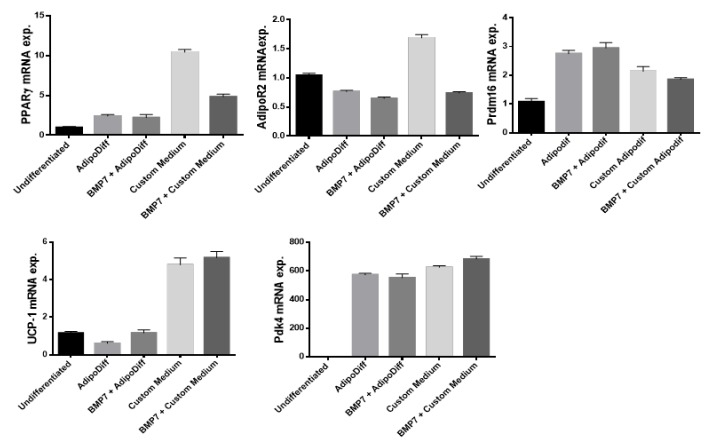
Confluent ADSCs were differentiated in different conditions, i.e., DMEM 10% FCS, AdipoDiff (Milteny Biotech), DMEM complemented with 10% FCS and 3.3 nM of BMP7, DMEM 10% complemented with 20 nM insulin (Sigma), 5 µM dexamethasone (Sigma), 125 µM indomethacin (Sigma), 1 nM triiodothyronine (T3), and 0.5 mM 3-isobutyl-1-methylxanthine (IBMX) in the absence or in the presence of 3.3 nM of hBMP7 for 15 days. Expression of adipose markers was analyzed by RT-PCR. ADSC derived beige cells were analyzed on Day 15 of differentiation. PPARγ, AdipoR, Prdm16, UCP-1, and Pdk4 mRNA expression levels were measured by RT-qPCR analysis. Data are the mean ± SEM (n = 5) and are expressed as relative expression ratios (ΔΔCt – fold increase).

Several protocols for ADSCs’ differentiation were tested. ADSCs were maintained for 15 days in commercial specific media (such as StemMACS AdipoDiff Media from Milteny Biotec), or in DMEM supplemented with hBMP7, or supplemented with adipo-growth factors and analyzed for the expression of adipocyte markers including PPARγ, AdipoR, Prdm16, UCP-1, and Pdk4 (Figure A2). Among all the tested conditions, the custom medium was found to be the most promising in terms of expression of brown tissue markers. Thus, in all the experiments listed below, confluent ADSCs were cultured for 15 days in basal medium complemented with insulin and dexamethasone to stimulate adipogenic differentiation, indomethacin, and 3-isobutyl-1-methylxanthine (IBMX) to modulate the expression of the PPARγ receptor and with triiodothyronine (T3) to increase UCP-1 expression. Figure 2a shows the morphological changes occurring in ADSCs upon differentiation. A clear sign of differentiation was the presence of small lipid droplets in differentiated ADSCs’ cytoplasm. Immunofluorescence and RT-PCR analyses for the expression of PPARγ, ACRP30, UCP-1, and PdK4 confirmed that ADSCs acquired brown cell molecular markers during the differentiation protocol (Figure 2b–d). Finally, we tested the metabolic activity of differentiated ADSCs using the Seahorse Cell Mito Stress Test. Although the basal oxygen consumption (OCR) of undifferentiated and differentiated ADSC seemed to be very similar, the maximal mitochondrial activity was significantly increased in differentiated ADSCs as demonstrated by the higher oxygen consumption measured by treating cells with the uncoupling agent FCCP. Furthermore, extracellular acidification increased in differentiated ADSCs compared to control ADSCs (Figure 2e,f). These data were confirmed by the ability of norepinephrine and isoproterenol to positively modulate the mitochondrial activity (Figure 2g) of differentiated ADSCs. Taken together, our results confirm that our protocol was suitable to drive ADSCs differentiation into ADSC derived beige cells.

### 3.3. ADSCs-Derived Beige Cells Show Pro-Angiogenic Properties

**Figure 3:**
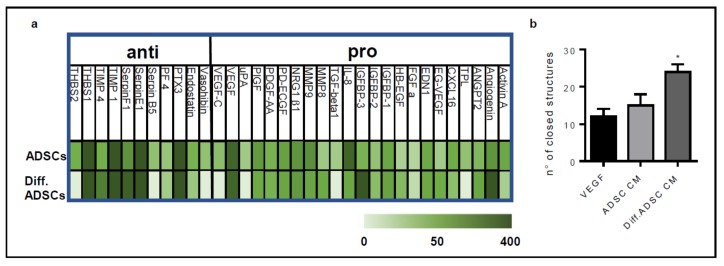
Conditioned medium of ADSC derived beige cells contains pro-angiogenic factors. Conditioned media were collected from confluent ADSCs and ADSC derived beige cells (Diff. ADSCs) cultured in basal medium for 48 h. (**a**) HUVEC cells (40,000 cells/cm^2^) were plated on reduced growth factor Cultrex and stimulated with conditioned medium obtained from ADSCs or ADSC derived beige cells. After 5 h, endothelial cell morphogenesis in terms of the formation of closed structures was examined using ImageJ software. Data are the mean ± SEM of three measurements per sample. * *p* < 0.05, Student’s *t*-test. (**b**) The Proteome Profiler Human Angiogenesis Antibody Array was used to detect angiogenesis related proteins in the conditioned medium of ADSCs and ADSC derived beige cells. Densitometry analysis of positive spot signals was normalized to positive and negative antibody array controls. Data are the mean of duplicate spots and expressed by color code (n = 2). Negative spots in both ADSCs and ADSC derived beige cell conditioned medium were not included in the analysis.

**Figure 4:**
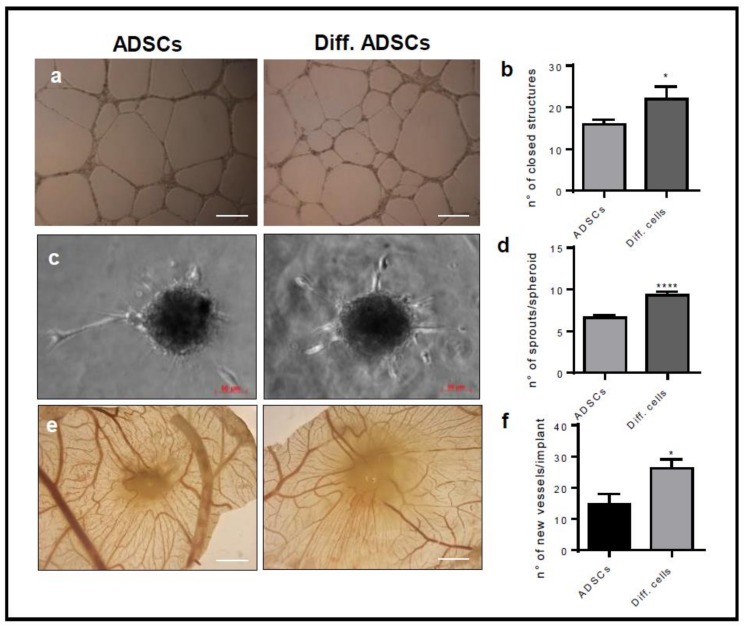
The differentiation into ADSC derived beige cells positively modulates the pro-angiogenic ability of ADSCs. (**a**) Confluent monolayers of ADSCs and ADSC derived beige cells (Diff.cells) were cultured in 24 wells. Two-hundred microliters of growth factor reduced Cultrex were added on the monolayers, and HUVEC cells were plated on gel. After 18 h, the formation of capillary-like structures was examined using a Zeiss Axiovert 200 M microscope (Scale bar 500 μm). Data are the mean ± SEM of three measurements per sample. * *p* < 0.05, Student’s *t*-test. (**b**) The number of closed structures. (**c**–**d**) HUVEC spheroids embedded in collagen gel and plated on an ADSC monolayer. The formation of radially growing sprouts was evaluated after 24 h of incubation. Data are the mean ± SEM of three independent experiments (* *p* < 0.05, **** *p* < 0.001, one-way ANOVA followed by Bonferroni’s test versus the control). (**e**–**f**) Alginate beads containing 3 × 10^4^ ADSCs or ADSC derived beige cells were implanted on the top of chick embryo chorioallantoic membranes (CAMs) at Day 11 of development. After 72 h, newly formed blood vessels converging toward the implant were counted in ovo at 5× magnification using an STEMI SR stereomicroscope equipped with an objective f equal to 100 mm with adapter ring 475,070 (Carl Zeiss) (Scale bar 2 mm). Data are the mean ± SEM (n = 6–8) (* *p* < 0.05, one-way ANOVA followed by Bonferroni’s test versus the control).

It is well known that mesenchymal stem cells support vessel recruitment and growth by producing and releasing several growth factors and chemokines. To assess whether ADSC derived beige cells maintain this pro-angiogenic activity, we evaluated their capacity to activate HUVECs in different angiogenic assays. To this, conditioned mediums were collected from ADSCs or ADSC derived beige cells cultured in basal medium for 48 h and tested in the tube formation assay. The conditioned medium of ADSC derived beige cells accelerated the morphogenesis of HUVE cells as demonstrated by the higher number of closed structures formed in 18 h compared to that induced by the conditioned medium of undifferentiated ADSCs (Figure 3a). Of note, VEGF-A, used as a positive control, exerted a pro-angiogenic effect, comparable to the one of the conditioned medium of undifferentiated ADSCs. The presence of pro-angiogenic factors in the conditioned medium of ADSC derived beige cells was confirmed by the human angiogenesis antibody array. For this, all proteins of conditioned medium were labelled with biotinylated antibodies and incubated on a spotted specific capture antibody membrane. The antibody array showed that ADSC derived beige cells continued to express and release in the extracellular environment high levels of several pro-angiogenic molecules including VEGF, FGF, PlGF, and PDGF (Figure 3b). To overcome the possible partial or total degradation of the soluble factors contained in the conditioned medium and to ensure a continuous release of soluble molecules, we set up an in vitro endothelial–ADSC co-culture system in which ADSCs were plated and differentiated for 15 days in the same well used for the co-culture. Then, 3D Cultrex gel was stratified in the well, and HUVECs were plated on it. Again, the ADSC derived beige cells accelerated the morphogenesis of HUVEC cells in terms of the number of closed structures. Of note, HUVECs cultured with undifferentiated ADSCs remained non-organized (Figure 4a,b). ADSC derived beige cells induced more sprouts from HUVEC-formed spheroids when embedded in 3D collagen gel in the co-culture system (Figure 4c,d). Importantly, in both co-culture systems, no physical interaction occurred between HUVEC and ADSCs. Furthermore, to mimic the co-culture system in an in vivo assay, ADSCs and ADSC derived beige cells were delivered on the top of the chick embryo chorioallantoic membrane (CAM) at Day 11 of development. In agreement with the in vitro results, ADSC derived beige cells supported the recruitment of host vessels from the surrounding tissues into the cell-enriched-3D scaffold on chick embryo CAM (Figure 4e,f).

### 3.4. Histogel Supports the Pro-Angiogenic Activity of ADSC-Derived Beige Cells

**Figure 5:**
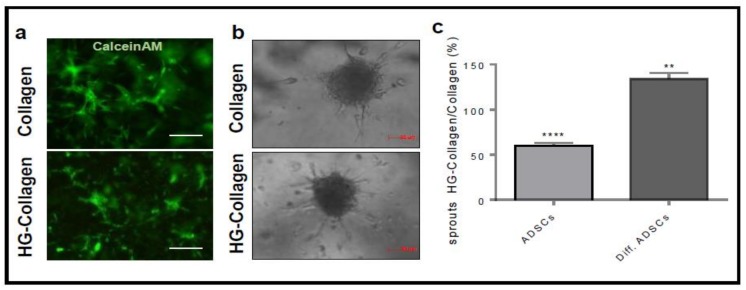
Histogel supports the angiogenic ability of ADSC derived beige cells. (**a**) ADSC derived beige cells were cultured in collagen or collagen:5% Histogel 1:1 for five days. Cells were photographed after 1 h of calcein-AM treatment (Scale bar 200 μm). (**b**,**c**) HUVEC spheroids were embedded in collagen gel or in collagen:5% Histogel 1:1 and plated on confluent monolayers of ADSCs and ADSC derived beige cells. The formation of radially growing sprouts was evaluated after 24 h of incubation. Representative figures (**b**). (**c**) describes the ratio of the number of sprouts formed from the endothelial cell spheroid co-cultured in 5% Histogel-collagen or in collagen gel with ADSCs or ADSC derived beige cells (ratio ± SEM of three independent experiments (**** *p* < 0.0001, ** *p* < 0.01, Student’s *t*-test versus the control).

Finally, we tested whether the pro-angiogenic ability of Histogel supports beige adipocyte potentiality. ADSC or ADSC derived beige cells were embedded in a co-culture system in a 3D bioscaffold containing a 1:1 ratio of Histogel/collagen on HUVEC sprout growth. Of note, the Histogel/collagen scaffold did not affect cell viability of either ADSCs or ADSC derived beige cells as demonstrated by the ability of their intracellular esterase to hydrolyze calcein-AM also for a long time (Figure 5a). ADSCs and ADSC derived beige cells were seeded and covered by HUVEC spheroids incorporated in the Histogel/collagen bioscaffold prototype. Results demonstrated that brown-like cells, also in the presence of the Histogel based bioscaffold, increased by 33% the angiogenic capacity of HUVEC cells as demonstrated by the higher number of newly formed sprouts (Figure 5b,c). ADSC derived beige cells synergized with the Histogel-collagen scaffold to induce angiogenesis as demonstrated by the ratio between endothelial sprouts formed in the 3D Histogel/collagen gel with respect to the 3D collagen gel co-cultured respectively with ADSCs or ADSC derived beige cells (Figure 5c).

## 4. Discussion

Obesity and its related disorders are mostly preventable conditions, but their treatment has proven to be a complex endeavor that has been mostly unsuccessful. The countermeasure usually suggested against overweightness and obesity is an increase in physical activity and/or a reduction of energy intake. A valid alternative to restore the normal physiological function of the human body could be the increase of energy consumption. BAT regulates the thermoregulation and metabolism, generating heat via non-shivering thermogenesis. In addition, beige adipocytes located in WAT also have thermogenic properties characterized by the expression of UCP1 [14]. Thus, the increase of brown tissue mass may represent a healthy and practical way to increase energy consumption, thus helping the individuals to control the weight balance [27]. For this purpose, here, we proposed the use of Histogel based bioscaffolds to promote the browning of adipose tissue. Histogel is a natural bioscaffold derived from porcine Wharton jelly. It is non-cytotoxic and non-hemolytic, and it does not induce inflammation [20]. Wharton jelly is a porous connective tissue found in umbilical cords of animal origin, forming a 3D spongy structure of fibrillar collagen and highly hydrated mucopolysaccharides, including hyaluronic acid proteoglycans [28,29]. The physiological function of Wharton jelly is to prevent the compression of the umbilical cord vessels. Decellularized Wharton jelly is a biocompatible scaffold with mechanical properties suitable to support cell adhesion, proliferation, and reorganization in 3D structures [30], appropriate for tissue engineering [31]. Furthermore, the low cost to obtain it and the “zero waste” material should not be underestimated. Histogel was isolated from porcine farming systems and by transforming them into high-end products with high potentiality for regenerative medicine and cosmetic devices by means of an eco-friendly processing. Here, we showed that Histogel promoted an angiogenic response both in vitro and in vivo. Histogel induced the migration, invasion, and reorganization into a tube-like structure of HUVEC cells and supported the recruitment of new blood vessels when implanted on the chorioallantoic membrane of chick embryos. Of note, the angiogenic process is necessary in tissue regeneration; thus, the pro-angiogenic ability of Histogel makes it a good bioscaffold for tissue engineering.

Biodegradability and porosity are critical features to keep in mind when designing bioscaffolds for tissue engineering. A good bioscaffold must remain in the tissue for the time necessary to support the engraftment of implanted cells, the recruitment of cells from surrounding tissue, and to sustain the metabolism of cells. Thus, the bioscaffold must remain in the host for a long time and not be degraded too quickly. To delay Histogel degradation and to achieve a longer permanence of Histogel in the host, we combined it with alginate or type I collagen gels. Both matrixes are currently used in the production of scaffolds for tissue engineering. Alginate is a polymer of mannuronic and glucuronic acid, extracted from brown algae [32]. Alginate has found numerous applications in biomedical sciences and engineering, such as controlled drug delivery for cartilage repair and regeneration [33]. Mammals lack alginase; thus, alginate results in being a non-degradable polymer. Therefore, in our protocol, alginate depolymerization was only dependent on the presence of monovalent ions in the microenvironment [34]. One critical drawback of alginate is its inherent lack of cell adhesivity [35]. In our system, this drawback was overcome by the high adhesivity of Histogel. Then, we tested Histogel-Type I collagen combinations. Type I collagen is a biodegradable material with remarkable water retention ability, low antigenicity, and cytocompatibility. Collagen is an efficient scaffold used for bone repair. It is also employed in regenerative medicine to promote regeneration of skin, cartilage, or ligaments [36]. The presented results supported that both Histogel based materials were biocompatible and provided good mechanical support, creating a scaffold suitable for cell adhesion and proliferation. Histogel based scaffolds allowed adhesion, migration, and endothelial cell reorganization and supported the adhesion and the survival of undifferentiated and differentiated ADSCs in vitro and in vivo.

Both histogel-alginate and Histogel-type I collagen gel scaffolds well supported on the one hand the differentiation of adipose tissue derived stem cells (ADSCs) into brown-like adipose cells expressing PPARγ, PdK4, and UCP-1 proteins and on the other hand the recruitment of blood vessels. The expression of specific adipose markers and the increase of OCR in differentiated ADSCs confirmed that our models well supported ADSCs differentiation. As expected, norepinephrine and isoproterenol increased the mitochondrial activity of ADSC derived beige cells [37,38].

Blood vessels are a necessary requirement to support BAT maintenance. In human adults, BAT consists of brown adipocytes, adipocyte progenitor cells, and blood vessels. BAT is a highly vascularized tissue located in the thorax in quantities inversely proportional to the size of the animal. Therefore, to increase brown adipose tissue or to promote the browning of WAT, it is essential to support the growth of new blood vessels. In our model, the ADSC derived beige cells produced and released in the microenvironment several pro-angiogenic factors, which, in association with the pro-angiogenic behavior of the Histogel based scaffolds, may contribute to supporting the vascularization of BAT.

The regeneration of BAT has been largely ignored in tissue engineering. Here, we proposed the use of biological scaffolds to support the proliferation and differentiation of the adipose tissue resident stem cells into a brown-like tissue and to allow the recruitment of vessels from the surrounding tissues. The employment of such devices will result in heavy BAT mass gain and efficient body weight loss. This would substantially improve the already existing applications in regenerative medicine and metabolic disease treatments.
